# Prevalence of depression and associated factors among critically ill pregnant women in Palestine

**DOI:** 10.1186/s40359-025-02698-w

**Published:** 2025-04-11

**Authors:** Mays Nabeel Aljoudi, Moath Abu Ejheisheh, Ibrahim Aqtam, Ahmad Ayed, Ahmad Batran

**Affiliations:** 1 Al-Ahli Hospital, Hebron, Palestine; 2 Faculty of Allied Medical Sciences, Department of Nursing, Ahliya University, Bethlehem, Palestine; 3 Department of Nursing, Ibn Sina College for Health Professions, Nablus University for Vocational and Technical Education, Nablus, Palestine; 4 Faculty of Nursing, Arab American University, Jenin, Palestine

**Keywords:** Prenatal depression, Critically ill pregnant women, Palestine, CUDOS scale, Risk factors

## Abstract

**Background:**

Depression during pregnancy poses significant challenges for both the mother and fetus, especially in cases where pregnancy complications become life-threatening. Recognizing the prevalence and risk factors associated with prenatal depression in critically ill pregnant women is essential, particularly in resource-limited settings like Palestine.

**Methods:**

A cross-sectional study was conducted among 304 critically ill pregnant women in eight Palestinian hospitals between January and March 2024. Data were collected through the Clinically Useful Depression Outcome Scale (CUDOS) and analyzed using SPSS Version 25. Descriptive statistics and multiple regression were applied to identify significant factors associated with depression severity.

**Results:**

The study found high levels of prenatal depression, with a mean CUDOS score of 55.72. Frequent crying (51%) and persistent fatigue (42.4% always, 41.1% sometimes) were common symptoms. Financial instability (*p* = 0.003), history of miscarriage (*p* = 0.005), unintended pregnancies (*p* = 0.001), and residing in rented housing (*p* = 0.004) were significant predictors, explaining a substantial variance in depression scores (adjusted R² = 0.56, *p* < 0.001).

**Conclusions:**

These results underscore the importance of routine mental health evaluations for critically ill pregnant women. Early detection and targeted interventions can improve outcomes for both mothers and their babies, offering essential insights for healthcare providers and policymakers.

**Practice implications:**

Incorporating mental health screening and support within maternal care programs in Palestine can help mitigate depression among high-risk pregnant women, improving maternal and fetal health outcomes.

## Introduction

Depression is a public health concern that seriously affects women during pregnancy. Prenatal depression carries risks for both the mother and fetus, including poor self-care, premature birth, and postpartum depression [1]. The physiological and hormonal changes in pregnancy may heighten symptoms of depression, leading to emotional, psychological, and physical challenges [2, 3].

Prenatal depression affects 10–15% of pregnancies worldwide, with higher rates in low-resource settings [4]. In Palestine, where access to healthcare is limited and socioeconomic hardships are common, stressors such as financial instability, poor social support, and life-threatening complications of pregnancy further heighten vulnerability to depression [5, 6]. These factors emphasize the urgency of addressing maternal mental health in such settings.

High-risk pregnancies, including conditions such as preeclampsia and severe hypertension, heighten the risks for depression [7, 8]. These complications lead to significant physical and emotional distress for both the mother and fetus. Although prenatal mental health is an established area of concern, research addressing prenatal depression in low-resource settings like Palestine is incomplete [9].

Prenatal depression is characterized by a loss of interest in everyday activities, ranging from mild to severe, significantly affecting daily functioning [10–13]. During pregnancy, depression impacts not only the mother but also the fetus, leading to impaired brain development, low birth weight, and increased neonatal morbidity [14]. Maternal depression can delay infant cognitive development, cause behavioral issues, and disrupt maternal-infant bonding. These disruptions may result in attachment disorders and long-term mental health challenges [15].

The prevalence of prenatal depression is higher in low-resource settings such as Palestine, with rates reaching 26% in some regions [3, 16]. Contributing factors include poverty, unintended pregnancies, and limited healthcare access, compounded by stressors like domestic violence and unemployment [4]. Women with high-risk pregnancies, such as those experiencing severe hypertension or preeclampsia, face heightened depression risks due to increased stress and intensive medical care needs [17, 18].

In conflict-affected regions, including the Middle East and North Africa (MENA), maternal mental health is particularly vulnerable [19]. Study from Saudi Arabia highlight how political instability, economic challenges, and trauma amplify prenatal depression. For example, women in Gaza exposed to both medical complications and political violence experience significantly higher depression rates [20].

Despite these risks, systematic screening for prenatal depression is lacking in low-resource settings. Evidence from countries like, Ethiopia and Brazil show that integrating mental health screening into prenatal care improves maternal and neonatal outcomes [21, 22]. Similar recommendations have been made for Palestine to use tools like the Clinically Useful Depression Outcome Scale (CUDOS) for early detection and intervention [23, 24]. The objective of this study is to assess the prevalence of prenatal depression among critically ill pregnant women in Palestine and identify associated sociodemographic and obstetric factors.

## Methodology

### Study design and setting

This was a cross-sectional study conducted between January and March 2024 in eight Palestinian hospitals: Muhammad Ali Al-Muhtaseb Hospital (Hebron), Alia Governmental Hospital (Hebron), Beit Jala Governmental Hospital (Bethlehem), Thabet Thabet Governmental Hospital (Tulkarm), Rafidia National Surgical Hospital (Nablus), Jenin Governmental Hospital (Jenin), Ramallah Medical Complex (Ramallah), and Al-Ahli Private Hospital. These hospitals varied in size and specialization, with capacities ranging from 150 to 500 beds, serving urban, rural, and refugee populations. Patients admitted to these hospitals typically included high-risk pregnancies, trauma cases, and individuals requiring specialized critical care.

### Study population

The study included critically ill pregnant women aged 18–45 years, attending prenatal care at the selected facilities, and presenting with clinically diagnosed depression as assessed by standardized diagnostic tools.

### Critical pregnancy condition definition

Critical pregnancy condition was defined as a life-threatening obstetric condition requiring intensive monitoring and medical intervention, including but not limited to severe hypertension, preeclampsia, eclampsia, and hemorrhagic complications. This term does not encompass psychological conditions such as depression but refers explicitly to physiological risks to maternal or fetal health.

### Sample selection and recruitment

**Fig. 1:**
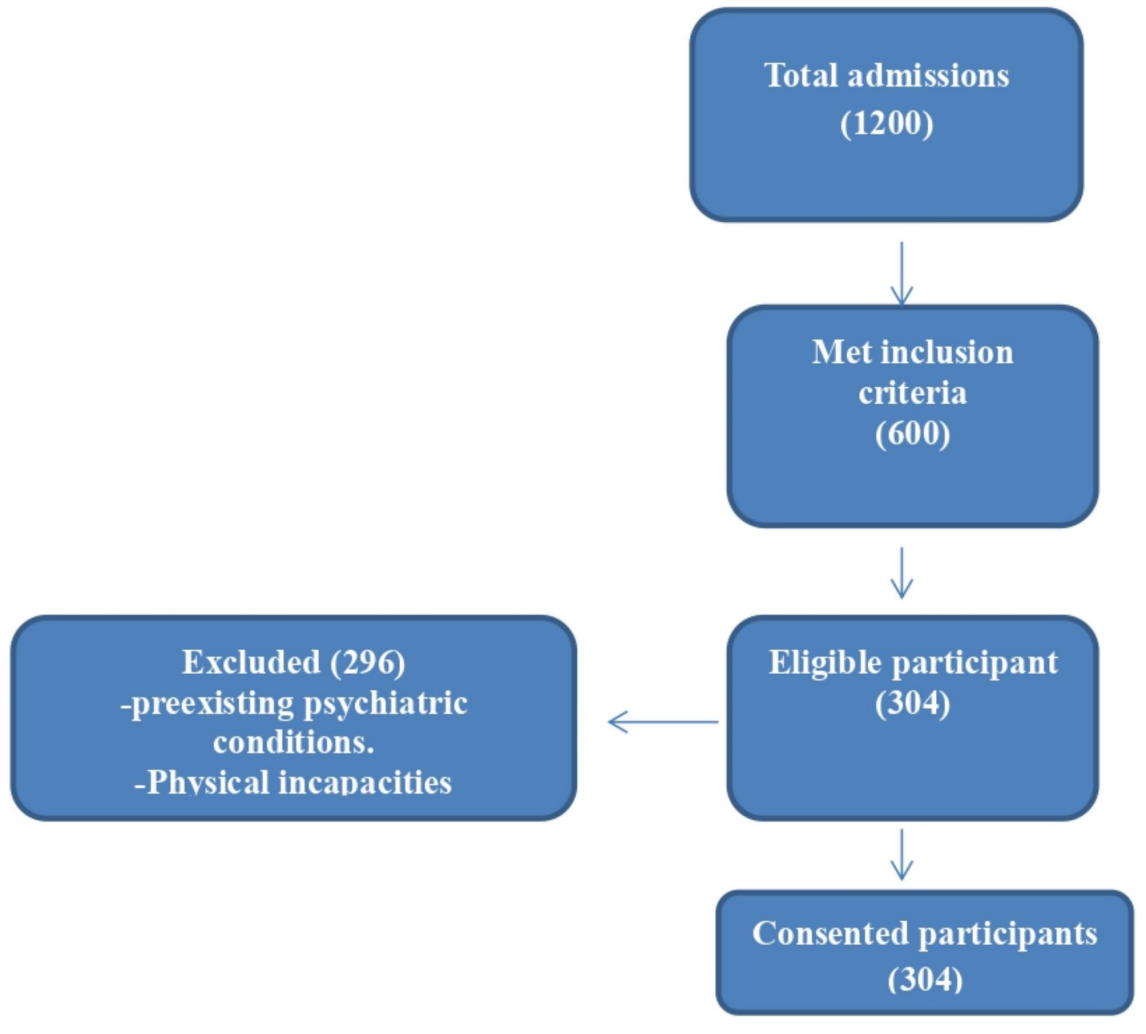
Flow chart of screening and recruitment process

A purposive sampling method was used to recruit 304 participants who met the inclusion criteria. A total of 1,200 admissions occurred during the study period. Of these, 600 patients were initially screened based on inclusion criteria, and 304 met the eligibility requirements (Fig. 1). The inclusion criteria were as follows: (1) pregnant women aged 18–45 years, (2) diagnosed with a critical illness requiring intensive medical care, (3) clinically assessed for depression using standardized tools, and (4) receiving prenatal care at one of the selected hospitals. The exclusion criteria included (1) women with pre-existing psychiatric disorders unrelated to depression, (2) patients unable to provide informed consent, and (3) those with cognitive impairments preventing questionnaire completion. Recruitment involved obtaining informed consent from stabilized patients. Participants were informed about their right to withdraw at any time without repercussions, addressing any perceived power dynamics due to their dependency on healthcare providers.

### Research instrument

A custom-designed questionnaire was used, divided into three sections:

1. **Sociodemographic data** (e.g., education, employment, and residence).
2. **Obstetric history** (e.g., previous pregnancies, complications, and miscarriages).
3. **Depression assessment using the Clinically Useful Depression Outcome Scale (CUDOS)** [24].

The CUDOS scale comprises 16 items, scored on a 5-point Likert scale, with a cut-off of 46 for severe depression. The questionnaire underwent a pilot test involving 10 participants from the same population, confirming clarity and yielding a Cronbach’s alpha of 0.91.

### Data collection

Data collection commenced following ethical approval from the Palestinian Ministry of Health and the relevant university ethics committee. Written informed consent was obtained from all participants prior to their enrollment in the study. Trained research assistants administered the questionnaires digitally using Google Forms and were present to ensure clarity without influencing responses. Participant anonymity and confidentiality were maintained, with data securely stored on password-protected servers accessible only to the principal investigators.

### Ethical considerations

Ethical approval was granted by the Palestine Ahliya University Research Ethics Committee (CAMS/CCNA/18/124). The study adhered to the Declaration of Helsinki, prioritizing participant welfare. Written informed consent was obtained from all participants.

### Data analysis

Data were analyzed using SPSS Version 25 [25]. Descriptive statistics were used to summarize demographic and clinical characteristics. Inferential statistics, including ANOVA, bivariate regression, and multiple linear regression, were employed to identify factors associated with depression, with a significance level set at *p* < 0.05.

### Limitations and Bias control

Efforts to minimize bias included ensuring participants had full insight during data collection and safeguarding data integrity through secure storage and limited access.

## Results

Out of 304 distributed questionnaires, all were completed, resulting in a 100% response rate. The demographic analysis revealed that the majority of participants, 133 (43.7%), were aged between 26 and 35 years, with 67 (22%) aged between 31 and 35 years. Most of the participants, 142 (46.6%), resided in villages, while 58 (19%) lived in refugee camps. Regarding education, 99 (32.6%) of the participants had completed high school, and 101 (33.2%) held a college diploma. Table 1 provides a detailed summary of participants’ demographic characteristics.

**Table 1:** Participant characteristics

Demographic Characteristics	*N*	%
Age		
Less than 20 years	55	18.1
21–25 years	61	20.1
26–30 years	66	21.7
31–35 years	67	22.0
36–40 years	38	12.5
More than 40 years	17	5.6
Residence		
Village	142	46.6
City	104	34.2
Refugee camp	58	19.0
Educational Level		
High School	99	32.6
College Diploma	101	33.2
Bachelor’s Degree	104	34.2

The analysis highlighted the prevalence of prenatal depression among critically ill pregnant women. Based on the CUDOS scoring criteria, 42.4% of participants reported experiencing severe depression, while 41.1% experienced moderate depression (Table 2). The mean CUDOS score of 55.72 reflects the overall severity of depression in the sample but does not directly explain the prevalence rates.

**Table 2:** Distribution of depression levels

Depression Severity	*N*	%
Severe depression (always)	129	42.4
Moderate depression (sometimes)	125	41.1
Mild depression	50	16.5

The most commonly reported symptoms of depression among participants included frequent crying and persistent tiredness. Specifically, 155 participants (51.0%) reported crying for no reason, while 129 participants (42.4%) indicated that they felt tired and discouraged ‘always.’ Additionally, 125 participants (41.1%) reported experiencing these feelings ‘sometimes.’ These findings, summarized in Table 3, may be influenced by the participants’ critical physical health conditions, which can exacerbate emotional distress and fatigue, further contributing to the reported depressive symptoms.

**Table 3:** The most commonly reported symptoms of depression among participants:

Symptom	Frequency (*N*)	Percentage (%)
Crying for no reason	155	51.0
Tiredness and discouragement (always)	129	42.4
Tiredness and discouragement (sometimes)	125	41.1

Significant associations were found between prenatal depression and certain obstetric factors, particularly among participants with a history of miscarriages (*p* < 0.001) and unintended pregnancies (*p* = 0.001). Specifically, 102 participants (33.6%) reported having experienced at least one miscarriage, and these individuals displayed significantly higher depression scores (*p* < 0.001). Additionally, 68 participants (22.4%) indicated that their pregnancies were unintended, and they exhibited higher levels of depression compared to those who reported planned pregnancies (*p* = 0.001). These findings underscore the impact of obstetric history on the mental health of pregnant women (Table 4).

**Table 4:** Obstetric factors and depression

Obstetric Factors	Depression Level	Total	*p*-value
History of Miscarriages	Yes	102	< 0.001**
	No	202	
Unintended Pregnancies	Yes	68	0.001**
	No	236	

Sociodemographic factors, particularly financial instability and living conditions, were more commonly observed among participants with higher levels of depression. Specifically, 88 (29.0%) participants reported experiencing financial difficulties, and this group exhibited significantly higher depression scores (*p* = 0.003). Furthermore, 120 (39.5%) participants who lived in rented accommodations also reported higher depression levels compared to those who owned their homes (*p* = 0.004). These findings highlight the critical role of socioeconomic conditions in influencing the mental health of individuals (Table 5).

**Table 5:** Sociodemographic factors and depression

Sociodemographic Factors	Depression Level	Total	*p*-value
Financial Instability	Yes	88	0.003**
	No	216	
Living in Rented Accommodations	Yes	120	0.004**
	No	184	

Table 6 presents the association between socio-demographic factors and depression levels during critical pregnancy among Palestinian women. The data were analyzed using one-way ANOVA to compare the mean depression scores across different categories, including participants’ age, husbands’ age, education level, spouses’ education level, and living conditions. The F-statistics and corresponding p-values indicate no statistically significant associations between these socio-demographic factors and depression levels, as all p-values exceeded the significance threshold of 0.05.

Mean depression scores varied by participants’ age, with those under 20 years scoring an average of 42.68 (SD = 16.06) and those aged 31–35 scoring 46.69 (SD = 16.79). Husbands’ age was also analyzed as a variable, with mean scores ranging from 34.54 (SD = 15.62) for husbands aged 21–25 to 42.17 (SD = 18.41) for husbands under 20. However, husbands were not directly assessed for depression in this study. Educational levels showed variations, with participants having less than a high school education scoring an average of 38.07 (SD = 18.18), while those with postgraduate education averaged 40.52 (SD = 14.39). Living conditions displayed differences, with participants from villages scoring an average of 35.80 (SD = 17.23) and those from cities scoring 43.21 (SD = 15.75). Despite these variations, none of the socio-demographic factors showed a significant association with depression levels during critical pregnancy.

**Table 6:** Association between Socio-demographic factors and depression scale during critical pregnancy among Palestinian women

Demographic data	Study participants (*n* = 304)				
	No.	Mean	SD	F	*P* value
Age/year				2.55	0.17
Less than 20 years old	55	42.68	16.06		
21–25 years old	61	43.61	15.51		
26–30 years old	66	44.28	13.93		
31–35 years old	67	46.69	16.79		
36–40 years old	38	40.20	14. 58		
More than 40 years old	17	42.00	16.29		
The age group of husbands				4.74	0.08
Less than 20 years old	11	42. 17	18.41		
21–25 years old	41	34. 54	15.62		
26–30 years old	46	37. 64	15.69		
31–35 years old	56	37.89	14.77		
36–40 years old	80	40.51	13.54		
More than 40 years old	70	40.08	13.47		
Highest level of education					
Less than high school	86	38.07	18.18	3.25	0.09
High school diploma	63	40.55	17.54		
Diploma	52	37.89	15.95		
Bachelor’s degree	88	40.51	15.75		
Postgraduate studies	15	40.52	14.39		
Highest level of education your spouse					
Less than high school	100	35. 38	12.81		
High school diploma	52	35.84	17.32	1.54	0.09
Diploma	48	38. 69	12.81		
Bachelor’s degree	84	40.55	15.77		
Postgraduate studies	20	43.25	14.30		
Living				1.04	0.14
Village	142	35.8	17.23		
Camp	58	38.07	12.74		
City	104	43.21	15.75		

As shown in Table 7, the Bivariate and Multilinear Regression Analysis identified significant predictors of depression among the study participants. Notably, financial instability (β = 1.85, 95% CI = 1.23 to 2.47, *p* < 0.001) and living conditions (β = 1.32, 95% CI = 0.75 to 1.89, *p* < 0.001) were strongly associated with higher depression scores. Participants experiencing financial difficulties (29.0%) and those living in rented accommodations (39.5%) reported significantly higher levels of depression compared to their counterparts. Additionally, pregnancy complications (β = 1.47, 95% CI = 0.82 to 2.12, *p* = 0.003) also emerged as a significant predictor, indicating that women facing life-threatening pregnancy-related conditions are at a greater risk of depression. Age was another factor, with older age showing a modest but significant association with depression (β = 0.12, 95% CI = 0.02 to 0.22, *p* = 0.021). In contrast, parity (β = 0.45, 95% CI = -0.03 to 0.93, *p* = 0.065) and education level (β = 0.39, 95% CI = -0.04 to 0.82, *p* = 0.074) did not show significant associations in the adjusted model. These findings emphasize the critical role of addressing socioeconomic and obstetric factors in mitigating depression risks among vulnerable populations.

The model accounted for 61.1% of the variance in depression scores (R-Square = 0.611), with an adjusted R-Square of 0.564. The overall regression model was statistically significant (F-value = 13.25, *p* = 0.001). These results underscore the urgent need for targeted interventions to address financial instability, inadequate living conditions, and pregnancy complications as part of comprehensive maternal mental health programs.

**Table 7:** Bivariate and multilinear regression analysis of factors associated with depression

Variable	Frequency (*n*)	Percentage (%)	Bivariate *p*-value	Regression Coefficient (β)	95% CI for β	*p*-value
Age (years)	-	-	0.045	0.12	0.02 to 0.22	0.021
Financial Instability	88	29.0	0.003	1.85	1.23 to 2.47	< 0.001
Living Conditions	120	39.5	0.004	1.32	0.75 to 1.89	< 0.001
Parity	-	-	0.081	0.45	-0.03 to 0.93	0.065
Pregnancy Complications	67	22.1	0.009	1.47	0.82 to 2.12	0.003
Education Level	-	-	0.125	0.39	-0.04 to 0.82	0.074

**Model Summary**: R-Square: 0.611, Adjusted R-Square: 0.564, F-value: 13.25 (*p* = 0.001)

## Discussion

This study sought to evaluate the prevalence and severity of depression among critically ill pregnant women in Palestine, as well as to identify the sociodemographic and obstetric factors associated with prenatal depression. The findings demonstrated a high prevalence of severe depression within this population, consistent with prior studies that highlight the susceptibility of women with high-risk pregnancies to depressive disorders [26–28]. Research from conflict-affected regions has reported elevated rates of perinatal depression, highlighting the role of environmental stressors in exacerbating mental health issues. For instance, a study conducted in Damascus, Syria, found that 28.2% of postpartum women exhibited probable depression, a prevalence higher than that reported in some other Arab countries. Notably, displacement due to the Syrian crisis was significantly associated with increased rates of postpartum depression [29]. Additionally, a study focusing on Syrian refugees and low-income Lebanese mothers accessing primary care in Lebanon reported high rates of maternal depression among Syrian refugees, further underscoring the impact of conflict-related stressors on perinatal mental health [30]. These findings emphasize the significant influence of conflict, displacement, and socioeconomic instability on the mental health of perinatal women in low-resource and conflict-affected settings.

The results of this study show that severe depression is widespread among critically ill pregnant women in Palestine, with significant correlations identified between depression and factors like financial instability, unintended pregnancies, and limited social support [12]. The implications of these findings warrant deeper exploration. For example, the role of economic instability in exacerbating depression among pregnant women could be further analyzed to identify specific mechanisms driving this relationship. Similarly, the impact of unintended pregnancies on mental health could be studied in more detail to develop targeted interventions for this group. Interestingly, while the study explored sociodemographic factors, no significant associations were found between these variables and depression levels, suggesting that characteristics like age, education, and living conditions may not be the primary drivers of depression in this context. This contrasts with some previous literature, which has linked certain demographic factors to mental health outcomes [29, 30]. For example, earlier studies have suggested that younger women may be at higher risk of depression, but the current findings indicate that such associations are less pronounced in this specific population.

Key obstetric factors emerged as strong predictors of depression, particularly complications with the current pregnancy and unplanned pregnancies, both of which were closely linked to higher depression scores [9]. These results align with earlier research, which has shown that complications and unintended pregnancies can heighten emotional distress and anxiety. Conversely, certain factors like smoking and employment status showed no significant correlation with depression, in line with studies that found minimal impact from these variables [31]. Additionally, the positive correlation between taking vitamins and iron supplements and lower depression scores reinforces prior findings on the importance of good nutrition and health behaviors in supporting mental health during pregnancy [24]. This suggests that adherence to health supplements may play a role in reducing depressive symptoms.

The prevalence of depression observed in this study mirrors the rates found in other low-resource settings, where prenatal depression is common among women with high-risk pregnancies [21, 32]. The findings highlight the critical need for mental health interventions in maternal care, especially in areas with limited healthcare resources and significant financial strain. Untreated prenatal depression can lead to adverse outcomes for both mother and child. Literature has consistently shown that depression is linked to poor self-care, which can negatively impact maternal health and increase the risk of negative outcomes for the fetus [31]. Addressing prenatal depression in critically ill pregnant women is therefore vital for improving both maternal and neonatal health.

The strengths of this study have been consolidated to provide a clear and cohesive overview. A key strength is its focus on a vulnerable group, critically ill pregnant women in Palestine, who are often overlooked in mental health research. The study also employed a substantial sample size and a validated tool (CUDOS), enhancing the reliability and validity of its findings. These elements contribute to the study’s ability to fill a significant gap in maternal mental health research in conflict-affected regions. However, some limitations must be acknowledged. The use of purposive sampling may limit the generalizability of the results to all pregnant women in Palestine, as the study included only those hospitalized in selected facilities [31]. Furthermore, the reliance on self-reported data could introduce bias, as participants may underreport or overreport their symptoms due to stigma or misunderstanding [15]. Additionally, the depression scale used in this study could not differentiate between symptoms arising from physical conditions, such as excessive tiredness, and those due to psychiatric causes, which may have influenced the interpretation of the results.

### Practical implications

The high prevalence of severe depression among critically ill pregnant women in Palestine underscores the necessity for routine mental health screening as part of maternal care in Palestinian hospitals. Healthcare providers should be trained to identify signs of prenatal depression and make appropriate referrals to mental health services. Incorporating mental health assessments into regular prenatal care could enable early detection and intervention, ultimately enhancing maternal health outcomes. Moreover, community-based programs that address financial instability and strengthen social support networks could help reduce the mental health burden in this population.

## Conclusion

In summary, this study underscores the high prevalence of prenatal depression among critically ill pregnant women in Palestine, highlighting the significant impact of socio-economic and obstetric factors on maternal mental health. Financial instability, unintended pregnancies, and the challenges of accessing adequate healthcare contribute to elevated levels of depression in this population. These findings not only emphasize the urgent need for routine mental health assessments within prenatal care services in Palestine but also provide important data that can inform global health policies. They highlight the critical importance of addressing maternal mental health in resource-limited and conflict-affected settings, offering insights applicable to other countries facing similar challenges.

### Recommendations

Future research should aim to expand the sample to include a broader range of healthcare settings and diverse patient demographics to improve the generalizability of the findings. Adapting the CUDOS scale to align more closely with the Palestinian socio-cultural context could enhance the accuracy of data on maternal mental health. Longitudinal studies would also offer valuable insights into the progression of prenatal depression over time, particularly among women facing socio-political adversity. Additionally, implementing intervention programs, such as mental health support and counseling within prenatal care, could be piloted in hospitals to improve maternal and fetal outcomes. Finally, comparative studies involving other conflict-affected regions could help identify universal and context-specific factors in managing prenatal depression effectively.

## Data Availability

No datasets were generated or analysed during the current study.
